# Assessing the Psychometric Properties of the Digital Behavior Change Intervention Engagement Scale in Users of an App for Reducing Alcohol Consumption: Evaluation Study

**DOI:** 10.2196/16197

**Published:** 2019-11-20

**Authors:** Olga Perski, Jim Lumsden, Claire Garnett, Ann Blandford, Robert West, Susan Michie

**Affiliations:** 1 Department of Behavioural Science and Health University College London London United Kingdom; 2 UK Centre for Tobacco and Alcohol Studies School of Experimental Psychology University of Bristol Bristol United Kingdom; 3 UCL Interaction Centre University College London London United Kingdom; 4 Department of Clinical, Educational and Health Psychology University College London London United Kingdom

**Keywords:** engagement, digital behaviour change interventions, mHealth, psychometrics, self-report scale, smartphone apps, excessive alcohol consumption

## Abstract

**Background:**

The level and type of engagement with digital behavior change interventions (DBCIs) are likely to influence their effectiveness, but validated self-report measures of engagement are lacking. The DBCI Engagement Scale was designed to assess behavioral (ie, amount, depth of use) and experiential (ie, attention, interest, enjoyment) dimensions of engagement.

**Objective:**

We aimed to assess the psychometric properties of the DBCI Engagement Scale in users of a smartphone app for reducing alcohol consumption.

**Methods:**

Participants (N=147) were UK-based, adult, excessive drinkers recruited via an online research platform. Participants downloaded the *Drink Less* app and completed the scale immediately after their first login in exchange for a financial reward. Criterion variables included the objectively recorded amount of use, depth of use, and subsequent login. Five types of validity (ie, construct, criterion, predictive, incremental, divergent) were examined in exploratory factor, correlational, and regression analyses. The Cronbach alpha was calculated to assess the scale’s internal reliability. Covariates included motivation to reduce alcohol consumption.

**Results:**

Responses on the DBCI Engagement Scale could be characterized in terms of two largely independent subscales related to experience and behavior. The experiential and behavioral subscales showed high (α=.78) and moderate (α=.45) internal reliability, respectively. Total scale scores predicted future behavioral engagement (ie, subsequent login) with and without adjusting for users’ motivation to reduce alcohol consumption (adjusted odds ratio [OR_adj_]=1.14; 95% CI 1.03-1.27; *P*=.01), which was driven by the experiential (OR_adj_=1.19; 95% CI 1.05-1.34; *P*=.006) but not the behavioral subscale.

**Conclusions:**

The DBCI Engagement Scale assesses behavioral and experiential aspects of engagement. The behavioral subscale may not be a valid indicator of behavioral engagement. The experiential subscale can predict subsequent behavioral engagement with an app for reducing alcohol consumption. Further refinements and validation of the scale in larger samples and across different DBCIs are needed.

## Introduction

Some level of engagement with digital behavior change interventions (DBCIs) is necessary for the effectiveness of such interventions [1]. However, observed levels of engagement with DBCIs are often considered too limited to support behavior change [2]. For example, a systematic review of Web-based health interventions found that approximately 50% of participants engaged with the interventions in the desired manner, with estimates varying between 10% and 90% across trials [3]. Studies conducted across different settings and target behaviors report a positive association of DBCI engagement and intervention effectiveness [4,5], suggesting that these variables may be linked via a dose-response function [1,6]. However, it is also plausible that individuals who are more successful in achieving change in the behavior targeted by the DBCI engage with DBCIs more [7] or that a limited amount of engagement is sufficient for bringing about meaningful change in some users (ie, “effective engagement”) [6]. Attempts have been made to characterize the function linking engagement with intervention effectiveness [1,7-9], but progress is hindered due to the use of different definitions and measures of engagement across studies.

The question of what it means for someone to be engaged with a DBCI has been of interest to psychologists and computer scientists alike. Broadly, psychologists have defined engagement as the extent of technology use, perceived as a proxy for participant exposure to a DBCI’s “active ingredients” or component behavior change techniques [10,11]. On the other hand, computer scientists have defined engagement as the subjective experience of “flow” or “immersion” that occurs during the human-computer interaction, characterized by focused attention, intrinsic interest, balance between challenge and skill, losing track of time and self-consciousness, and transportation to a “different place” [12,13]. After having conducted a systematic, integrative literature review of the psychology and computer science literatures [7] in addition to in-depth interviews with potential DBCI users, our interdisciplinary research team proposed the following working definition of engagement: “[Engagement with a DBCI is] a state-like construct which occurs each time a user interacts with a DBCI, with two behavioral (ie, amount and depth of use) and three experiential (ie, attention, interest and enjoyment) dimensions [14].”

We hence theorized that two behavioral (amount and depth of use) and three experiential (attention, interest, and enjoyment) dimensions are necessary and sufficient conditions for someone to be engaged with a DBCI. Although similar, engagement with DBCIs is thought to be conceptually distinct from both “flow” and pure technology usage. Although several measures of flow, immersion, and technology usage are available for use (for overviews, see [7,14,15]), an instrument that quantifies the intensity of behavioral and experiential engagement is lacking. For a quantitative scale of engagement to be useful for researchers, practitioners, and developers, it should be able to predict key variables of interest such as future engagement, knowledge acquisition, or intervention effectiveness. In addition, although a number of usage metrics derived from log-data are typically used to capture the intensity of behavioral engagement [15-17], a validated measure of engagement, which captures both the experiential and behavioral dimensions of engagement and could be easily administered without the need to access and process the DBCI’s raw data, would be useful. The DBCI Engagement Scale was developed to fill this gap [14].

As part of the scale development process (described in detail in [14]), a pool of initial scale items was developed by the interdisciplinary research team in addition to two “best bets” for a short measure of engagement. Lay and expert respondents were then asked to classify the initial scale items into one of six categories (ie, *amount of use*, *depth of use*, *interest*, *attention*, *enjoyment*, plus an *unclassified* category) to examine the scale’s content validity. The first psychometric evaluation of the 10-item DBCI Engagement Scale was conducted in a sample of adult excessive drinkers who had voluntarily downloaded a freely available, evidence-informed app—*Drink Less—*for reducing their alcohol consumption [14]. Results indicated that the behavioral and experiential indicators of engagement may resolve to a single dimension. However, fewer than 5% of eligible users completed the scale during the study, and a sensitivity analysis indicated that the analytic sample was biased toward highly engaged users.

Studying engagement in real-world settings is notoriously difficult, as highly engaged users are more likely to respond to research surveys [18], potentially biasing results. Moreover, evidence suggests that motivation to change the target behavior is consistently associated with the frequency of behavioral engagement, such as the total number of logins [19,20]. Although motivation to change is a key predictor of engagement, it is neither a necessary nor a sufficient condition for someone to be engaged with a DBCI. For example, a user with low motivation to reduce their alcohol consumption might be intrigued by the design of a specific app, engage with its content, and subsequently become motivated to drink less. Therefore, to better study the dimensional structure of engagement, we considered it important to adjust for motivation to change in our analyses, thus separating the state of engagement from confounding motivations. This study aimed to evaluate the DBCI Engagement Scale in a sample of users recruited via an online research platform in order to address the following research questions:

1. What is the factor structure of the DBCI Engagement Scale? (construct validity)
2. Is the DBCI Engagement Scale internally reliable? (internal reliability)
3. Are total scale scores positively associated with objectively recorded amount of use and depth of use? (criterion validity)
4. Do total scale scores predict future behavioral engagement (ie, subsequent login), with and without adjustment for motivation to reduce alcohol consumption? (predictive validity)
5. Do two best bets for a short measure of engagement predict future behavioral engagement, with and without adjustment for motivation to reduce alcohol consumption? (predictive validity)
6. Does a model including the objectively recorded behavioral and the self-reported experiential indicators of engagement account for more variance in future behavioral engagement (ie, subsequent login) compared with a model including only the objectively recorded behavioral indicators of engagement? (incremental validity)
7. Are total scale scores significantly associated with scores on the Flow State Scale? (divergent validity)

## Methods

The preregistered study protocol can be found in the Open Science Framework [21]. Ethical approval was granted by University College London’s Computer Science Departmental Research Ethics Chair (Project ID: UCLIC/1617/004/Staff Blandford HFDH).

### Inclusion Criteria

Participants were eligible to take part in the study if they were aged ≥18 years; reported an Alcohol Use Disorders Identification Test (AUDIT) score ≥8, indicating excessive alcohol consumption [22]; were residing in the United Kingdom; owned an iPhone capable of running iOS 8.0 software (ie, an iPhone 4S or later models); and were willing to download and explore an app for reducing alcohol consumption.

### Sampling

Participants were recruited via the online research platform Prolific [23]. Individuals who take part in research via online platforms are primarily motivated by the financial incentives and are not necessarily interested in health behavior change [24]. Therefore, we expected that Prolific would enable us to recruit a sample of users with different levels of motivation to change. We did not, however, expect to recruit a sample that is representative of the general population of excessive drinkers in the United Kingdom.

### Sample Size

No formal sample size calculation was performed. Based on the psychometric literature, a 25:1 participant-to-item ratio (ie, a total of 250 participants) was considered desirable [25].

### Measures

To determine eligibility and describe the sample, data were collected on age; sex (female or male); type of work (manual, nonmanual, or other); patterns of alcohol consumption, measured by the AUDIT [22]; motivation to reduce alcohol consumption, measured by the Motivation To Stop Scale [26-28]; country of residence (United Kingdom or other); iPhone ownership (yes or no); and willingness to download and explore an alcohol reduction app (yes or no).

For eligible participants who downloaded and explored the *Drink Less* app, data were collected on location during first use of the app (home, work, vehicle, public transport, restaurant/pub/café, other’s home, other, or can’t remember) and the 10-item DBCI Engagement Scale [14], which captures momentary behavioral (ie, amount, depth of use) and experiential (ie, attention, interest, enjoyment) engagement with DBCIs (Textbox 1). A detailed account of how the scale items were developed and tested in a group of experts and nonexperts can be found in a previous study [14].

Data were also collected on the below variables, which were used to test the scale’s criterion, predictive, incremental, and divergent validity.

**The DBCI Engagement Scale.**

Please answer the following questions with regard to your most recent use of the *Drink Less* app.

How strongly did you experience the following?

1. Interest

2. Intrigue

3. Focus

4. Inattention

5. Distraction

6. Enjoyment

7. Annoyance

8. Pleasure *(Measured on a 7-point scale with end- and middle-points anchored: “not at all,” “moderately,” and “extremely”)*

9. How much time (in minutes) do you roughly think that you spent on the app? *(Enter free text)*

10. Which of the app’s components do you remember visiting? (You can select multiple options)

a) Calendar *(coded by the researchers as “Self-Monitoring/Feedback”)*

b) Create and view goals *(coded as “Goal Setting”)*

c) What has and hasn’t worked *(coded as “Self-Monitoring/Feedback”)*

d) Create and view action plans *(coded as “Action Planning”)*

e) Your hangover and you *(coded as “Self-Monitoring/Feedback”)*

f) Review your drinking *(coded as “Normative Feedback”)*

g) Dashboard *(coded as “Self-Monitoring/Feedback”)*

h) Game *(coded as “Cognitive Bias Re-Training”)*

i) Drink + me *(coded as “Identity Change”)*

j) Useful information *(coded as “Other”)*

k) Other *(coded as “Other”)*

l) Can’t remember *(coded as “Other”)*

*Indexed as a proportion of available modules (eg, 5/7×100=71.4).*

#### Construct, Criterion, and Incremental Validity

A record of the number of app screens viewed was kept during participants’ first login session to derive the objectively recorded *amount of use* and *depth of use*, which were used to test the scale’s construct, criterion, and incremental validity. The screen view records were stored in an online database (*NodeChef*) and extracted using the free python library *pandas*. The variable *amount of use* was derived by calculating the time spent (in seconds) during participants’ first login session. The variable *depth of use* was derived by calculating the number of app components visited during participants’ first login session, indexed as a proportion (0-100) of the number of available components within the *Drink Less* app (ie, Goal Setting, Self-monitoring/Feedback, Action Planning, Normative Feedback, Cognitive Bias Re-Training, Identity Change, Other [29]).

#### Predictive Validity

A record of the number of app screens viewed was also kept over the next 14 days to derive the variable *subsequent login*, which was used to test the scale’s predictive validity. A subsequent login (yes vs no) was defined as a new screen view following at least 30 minutes of inactivity [30]. As health apps are likely to be abandoned after users’ first login [31,32], the authors theorized that a useful measure of engagement should be able to distinguish between users who are likely to return to an app.

Two items that represented the authors’ *best bets* for a short measure of engagement (ie, “How much did you like the app?” and “How engaging was the app?”) were developed by the study team and used to test whether a short measure of engagement had superior predictive validity compared with the scale in its entirety. These items were not explicitly drawn from published self-report scales.

#### Divergent Validity

Two items from the Flow State Scale [33] were used to test the scale’s divergent validity. We selected two items that were previously found to load most strongly onto the general *flow* factor (ie, “When using *Drink Less*, the way time passed seemed to be different from normal,” “When using *Drink Less,* I was not worried about what others may have been thinking of me”). Although there is some overlap in the experiential indicators of the states of engagement and flow (ie, focused attention, interest), the study team theorized that users do not necessarily experience *loss of time and consciousness* or *balance between challenge and skill* when engaging with a DBCI. Assessing whether users can be engaged without necessarily being in a state of flow was therefore considered a useful test of the scale’s divergent validity. The Flow State Scale has previously been applied in the context of digital gaming [34].

### Procedure

Interested participants were identified via the recruitment platform, Prolific, and received a compensation of £0.50 for completing the screening questionnaire, hosted by Qualtrics survey software (Provo, Utah). Eligible participants were invited via Prolific’s internal email system and asked to download the *Drink Less* app from the Apple App Store. Participants were instructed to explore the *Drink Less* app in the way that they would explore any new app and were told that the researchers would monitor their app usage to assess what content they were interested in. For technical reasons, participants were told that they had to select the option *Interested in drinking less alcohol* when asked about why they were using the *Drink Less* app and to enable the push notifications. When clicking on the phone’s home button after having finished exploring the app, participants received a push notification with a link to the study survey. Participants were subsequently asked to enter their Prolific identification number, which enabled the researchers to match participants’ survey responses to their app screen views. Participants who initiated but did not complete the study survey (as indicated by their response status on Prolific’s platform, which was either labelled “Timed out” or “Returned submission”) were sent one reminder message. On completing the task, participants were paid £1.25.

### Data Analysis

All analyses were conducted in SPSS version 20.0 (IBM Corporation, Armonk, New York). The assumptions for parametric tests were assessed (ie, normality of the distribution of residuals) and when violated, normalization was applied (ie, *z*-score normalization of positively skewed data). Descriptive statistics (eg, mean, range, variance) were calculated for each scale item and the criterion variables of interest to determine suitability for factor analysis.

#### Construct Validity

It was hypothesized that a five-factor solution (ie, *amount of use*, *depth of use*, *attention*, *interest*, *enjoyment*) would provide the best fit of the observed data [14]. A series of exploratory factor analyses (EFAs) using principal axis factoring estimation and oblique rotation was conducted. The inspection of Cattell’s scree plots and the Kaiser criterion (ie, factors with eigenvalues >1) was used to determine the number of factors to retain [25]. First, we tested the fit of a solution including the self-reported items. This was compared with a solution including a combination of the self-reported indicators of experiential engagement and the objectively recorded indicators of behavioral engagement (ie, objective *amount of use* and *depth of use*).

#### Internal Reliability

Internal consistency reliability was assessed by calculating the Cronbach alpha. A large coefficient (ie, =.70 or above) was interpreted as evidence of strong item covariance [35].

#### Criterion Validity

Criterion validity was assessed by calculating the Pearson correlation coefficient for the relationship between participants’ automatically recorded app screen views from their first login (ie, objective *amount of use* and *depth of use*) with their self-reported *amount of use* and *depth of use* and their total scale scores.

#### Predictive Validity

The variable *subsequent login* was regressed onto participants’ total scale scores, with and without adjustment for motivation to reduce alcohol consumption.

The variable *subsequent login* was also regressed onto each of the two *best bets* for a short measure of engagement (ie, “How engaging was the app?” and “How much did you like the app?”), with and without adjustment for motivation to reduce alcohol consumption.

#### Incremental Validity

Incremental validity was assessed in two steps. First, we assessed the variance accounted for in the variable *subsequent login* by the objectively recorded indicators of behavioral engagement. This was compared with the variance accounted for in the variable *subsequent login* after adding the self-reported indicators of experiential engagement to the objectively recorded indicators of behavioral engagement.

#### Divergent Validity

Divergent validity was assessed by calculating the Pearson correlation coefficient for the relationship between each of the two indicators of the state of flow from the Flow State Scale [33] and the overall measure of engagement.

## Results

### Participants

**Figure 1:**
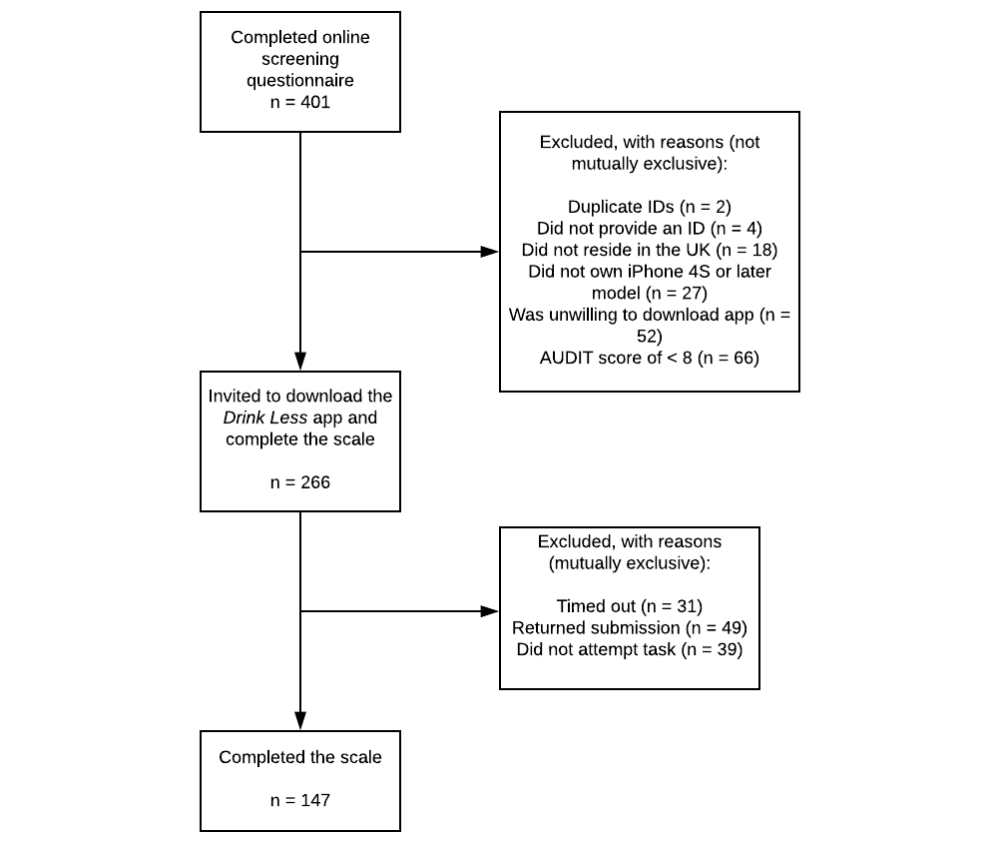
Participant flow chart. AUDIT: Alcohol Use Disorders Identification Test; ID: identification.

During the study period (31 days; July 23, 2018 to August 22, 2018), 401 participants completed the online screening survey, of which 266 were eligible to take part. Of these, 147 (55%) participants downloaded the *Drink Less* app and completed the task (Figure 1). Due to funding restrictions, we were unable to extend the recruitment beyond this time point. The desired target sample size of 250 participants was hence not achieved. Participants’ demographic and drinking characteristics are reported in Table 1. We did not detect any significant differences between eligible participants who did and did not complete the task on the demographic characteristics assessed.

**Table 1:** Participants’ demographic and drinking characteristics.

Demographic characteristics			Completed scale (n=147)	Eligible but did not complete scale (n=119)	*P* value^*a*^
Female gender, n (%)			97 (66)	71 (60)	.29
**Type of work, n (%)**					**.57**
	Manual, n (%)		19 (13)	16 (13)	
	Nonmanual, n (%)		89 (61)	78 (66)	
	Other, n (%)		39 (27)	25 (21)	
Age (years), mean (SD)			34.4 (10.4)	36.6 (11.8)	.11
**Drinking characteristics**					
	**Motivation To Stop Scale, n (%)**				**.08**
		I don’t want to cut down on drinking alcohol	14 (10)	26 (22)	
		I think I should cut down on drinking alcohol but I don’t really want to	43 (29)	25 (21)	
		I want to cut down on drinking alcohol but I haven’t thought about when	19 (13)	17 (14)	
		I really want to cut down on drinking alcohol but I don’t know when I will	17 (12)	11 (9)	
		I want to cut down on drinking and hope to soon	23 (16)	17 (14)	
		I really want to cut down on drinking alcohol and intend to in the next 3 months	11 (7)	4 (3)	
		I really want to cut down on drinking alcohol and intend to in the next month	20 (14)	19 (16)	
	Alcohol Use Disorders Identification Test, mean (SD)		15.4 (5.1)	14.2 (5.7)	.07

^a^Differences between groups were assessed using chi-square tests or *t* tests, as appropriate.

### Descriptive Statistics

Descriptive statistics for the scale items are reported in Table 2. The majority of participants completed the scale at home (118/147, 80.3%) or work (19/147, 12.9%). To account for the observed skewness, z-score normalization was applied to the 10-scale items and the two items used for testing the scale’s criterion validity. Inter-item correlations of the normalized scale items are reported in Table 3.

### Scale Evaluation

#### Construct Validity

The Keiser-Meier Olkin Test of Sampling Adequacy (0.70) and the Bartlett Test of Sphericity (*P*<.001) indicated that data were suited for factor analysis. Three EFA solutions were tested to arrive at a best-fitting solution.

**Table 2:** Descriptive statistics for the scale items (N=147).

Statistic		Range	Mean (SD)	Variance	Skewness	Kurtosis
**DBCI^a^ Engagement Scale Items**						
	1. “How strongly did you experience interest?”	2-7	5.30 (1.09)	1.18	–0.30	0.06
	2. “How strongly did you experience intrigue?”	1-7	5.39 (1.27)	1.61	–0.85	0.50
	3. “How strongly did you experience focus?”	2-7	5.31 (1.18)	1.40	–0.56	0.14
	4. “How strongly did you experience inattention?”^b^	1-7	5.61 (1.33)	1.76	–1.24	1.47
	5. “How strongly did you experience distraction?”^b^	1-7	5.47 (1.45)	2.10	–1.12	0.86
	6. “How strongly did you experience enjoyment?”	1-7	4.46 (1.44)	2.07	–0.10	–0.48
	7. “How strongly did you experience pleasure?”	1-7	3.56 (1.64)	2.67	0.36	–0.70
	8. “How strongly did you experience annoyance?”^b^	1-7	5.59 (1.39)	1.93	–1.09	1.08
	9. “Which of the app’s components did you visit?”	14.29-100.00	58.70 (22.00)	484.01	–0.12	–0.67
	10. “How much time do you roughly think that you spent on the app?” (seconds)	120-1200	520.82 (237.21)	56,267.82	0.93	0.96
**Variables used to test the scale’s construct, criterion, and incremental validity**						
	11. Objectively recorded depth of use	28.57-100.00	66.66 (20.50)	420.28	–0.23	–0.85
	12. Objectively recorded amount of use (seconds)	95.00-3,571.00	409.45 (360.71)	130,116.72	5.13	40.34
**Items used to test the scale’s divergent validity**						
	13 “When using *Drink Less*, the way time passed seemed different from normal.”	1-5	2.76 (0.79)	0.62	0.11	0.10
	14. “When using *Drink Less*, I was not worried about what others may have been thinking about me.”	1-5	3.34 (1.16)	1.35	–0.24	–1.11
**Variables/items used to test the scale’s predictive validity**						
	15. “How much did you like the app?”	1-7	5.14 (1.29)	1.66	–0.80	0.82
	16. “How engaging was the app?”	1-7	5.20 (1.17)	1.37	–0.65	0.66
	17. Subsequent login (yes vs no), n (%)	67 (46)	N/A^c^	N/A	N/A	N/A

^a^DBCI: digital behavior change intervention.

^b^Values were reverse scored prior to the calculation of descriptive statistics.

^c^Not applicable.

**Table 3:** Inter-item correlation matrix (N=147).

DBCI Engagement Scale items		1^a^	2^b^	3^c^	4^d,e^	5^e,f^	6^g^	7^h^	8^e,i^	9^j^	10^k^	11^l,m^	12^m,n^
**1. Interest**		**1**											
	*P* value	N/A^o^											
**2. Intrigue**		**0.44**	**1**										
	*P* value	<.001	N/A										
**3. Focus**		**0.65**	**0.46**	**1**									
	*P* value	<.001	<.001	N/A									
**4. Inattention^e^**		**0.18**	**0.10**	**0.31**	**1**								
	*P* value	.027	.21	<.001	N/A								
**5. Distraction^e^**		**0.18**	**0.12**	**0.28**	**0.43**	**1**							
	*P* value	.026	.16	.001	<.001	N/A							
**6. Enjoyment**		**0.48**	**0.31**	**0.44**	**–0.05**	**–0.15**	**1**						
	*P* value	<.001	<.001	<.001	.59	.071	N/A						
**7. Pleasure**		**0.19**	**0.09**	**0.15**	**–0.19**	**–0.24**	**0.54**	**1**					
	*P* value	.025	.30	.079	.019	.003	<.001	N/A					
**8. Annoyance^e^**		**0.28**	**0.15**	**0.37**	**0.27**	**0.24**	**0.29**	**0.12**	**1**				
	*P* value	.001	.07	<.001	.001	.004	<.001	.16	N/A				
**9. Which of app’s components**		**0.18**	**0.00**	**0.06**	**0.13**	**–0.03**	**0.19**	**0.19**	**.13**	**1**			
	*P* value	.028	.97	.469	.11	.75	.019	.02	.13	N/A			
**10. How much time spent**		**0.10**	**0.10**	**–0.03**	**0.08**	**0.11**	**0.15**	**0.33**	**0.09**	**0.29**	**1**		
	*P* value	.23	.22	.681	.33	.18	.07	<.001	.31	<.001	N/A		
**11. Objective depth of use^m^**		**0.13**	**0.11**	**0.15**	**0.18**	**0.01**	**0.11**	**–0.01**	**0.24**	**0.51**	**0.16**	**1**	
	*P* value	.12	.18	.069	.03	.90	.20	.94	.003	<.001	.051	N/A	
**12. Objective amount of use^m^**		**0.31**	**0.18**	**0.28**	**0.16**	**0.06**	**0.25**	**0.00**	**0.19**	**0.10**	**0.10**	**0.52**	**1**
	*P* value	<.001	.03	.001	.047	.49	.002	.97	.02	.22	.23	<.001	N/A

^a^Interest.

^b^Intrigue.

^c^Focus.

^d^Inattention.

^e^Values were reverse scored prior to analysis.

^f^Distraction.

^g^Enjoyment.

^h^Pleasure.

^i^Annoyance.

^j^Which of the app’s components.

^k^How much time spent.

^l^Objective depth of use.

^m^Variables used to test the scale’s construct, criterion, and incremental validity.

^n^Objective amount of use.

^o^Not applicable.

#### Solution 1

An EFA with oblique rotation was conducted. The eigenvalues indicated that a three-factor solution, accounting for 61.2% of the variance, was most appropriate (Table 4). The loadings indicated that the second factor comprised two of the negatively worded indicators (ie, items 4 and 5). The third factor comprised the two behavioral indicators (ie, items 9 and 10) and one of the experiential indicators (ie, item 7), which made little theoretical sense [14]. The loading of item 8 (also a negatively worded item) onto factor 1 was modest. Therefore, the negatively worded items (ie, items 4, 5, and 8) and item 7 were discarded prior to conducting a second EFA.

#### Solution 2

A subsequent EFA with oblique rotation indicated that a two-factor solution accounted for 62.4% of the variance (Table 4). The experiential indicators loaded clearly onto factor 1, and the behavioral indicators loaded clearly onto factor 2, with no cross-loadings (ie, items that load at 0.32 or higher on two or more factors) [25]. The two latent factors were labelled *Experiential Engagement* and *Behavioral Engagement*, respectively.

#### Solution 3

An EFA with oblique rotation using a combination of the self-reported experiential indicators (ie, items 1, 2, 3, and 6) and the automatically recorded behavioral indicators (ie, items 11 and 12) suggested a two-factor solution, which accounted for 65.7% of the variance. The experiential indicators loaded clearly onto factor 1, and the behavioral indicators loaded clearly onto factor 2 (Table 4).

Solution 2 was selected for use in the subsequent reliability and validity analyses, as it contained only the self-reported items and provided a similarly good fit of the data as Solution 3. A total scale score was calculated for each participant, with equal weight given to each of the retained items (ie, items 1, 2, 3, 6, 9, and 10).

**Table 4:** Factor loadings of the DBCI Engagement Scale in exploratory factor analyses.

Scale Items	Solution 1^a^			Solution 2^b^		Solution 3^c^	
	Factor 1	Factor 2	Factor 3	Factor 1	Factor 2	Factor 1	Factor 2
1. Interest	0.75^d^	0.14	0.25	0.80^d^	0.26	0.82^d^	0.28
2. Intrigue	0.51^d^	0.09	0.11	0.55^d^	0.09	0.55^d^	0.18
3. Focus	0.87^d^	0.28	0.09	0.83^d^	0.02	0.80^d^	0.27
4. Inattention^e^	0.25	0.61^d^	0.14	N/A^f^	N/A	N/A	N/A
5. Distraction^e^	0.21	0.68^d^	0.06	N/A	N/A	N/A	N/A
6. Enjoyment	0.66^d^	–0.35	0.43^d^	0.57^d^	0.31	0.57^d^	0.23
7. Pleasure	0.31	–0.48	0.56^d^	N/A	N/A	N/A	N/A
8. Annoyance^e^	0.41^d^	0.23	0.25	N/A	N/A	N/A	N/A
9. Which of app’s components	0.16	0.01	0.43^d^	0.15	0.55	N/A	N/A
10. How much time spent	0.10	0.03	0.64^d^	0.09	0.53	N/A	N/A
11. Objective depth of use	N/A	N/A	N/A	N/A	N/A	0.37	0.77^d^
12. Objective amount of use	N/A	N/A	N/A	N/A	N/A	0.18	0.68^d^

^a^Exploratory factor analysis with oblique rotation, including items 1-10.

^b^Exploratory factor analysis with oblique rotation, including items 1, 2, 3, 6, 9, and 10.

^c^Exploratory factor analysis with oblique rotation, including items 1, 2, 3, 6, 11, and 12.

^d^Values with factor loadings ≥0.40.

^e^Values were reverse scored prior to analysis.

^f^Not applicable.

#### Internal Reliability

The internal consistency of the overall measure was 0.67, indicating moderate internal reliability [35]. The *Experiential Engagement* subscale had an internal consistency of 0.78, while the *Behavioral Engagement* subscale had an internal consistency of 0.45. Both subscales were significantly correlated with the measure overall (*r*_145_=0.90, *P*<.001 and *r*_145_=0.56, *P*<.001, respectively). However, the subscales were not significantly correlated with each other (*r*_145_=0.15, *P*=.07).

#### Criterion Validity

Total scale scores were significantly correlated with objectively recorded *depth of use* (*r*_145_=0.32, *P*<.001) and objectively recorded *amount of use* (*r*_145_=0.33, *P*<.001). Self-reported *depth of use* was significantly correlated with objectively recorded *depth of use* (*r*_145_=0.51, *P*<.001). Self-reported *amount of use* was not significantly correlated with objectively recorded *amount of use* (*r*_145_=0.10, *P*=.23).

#### Predictive Validity

Results from the predictive validity analyses are presented in Table 5. In the unadjusted analysis, total scale scores were significantly associated with future behavioral engagement, ie, the variable *subsequent login* (odds ratio [OR]=1.15, 95% CI 1.05-1.27, *P*=.01). The association remained significant in the model adjusting for motivation to reduce alcohol consumption (adjusted OR [OR_adj_]=1.14, 95% CI 1.03-1.27, *P*=.01).

As the two subscales (ie, *Behavioral Engagement* and *Experiential Engagement*) were not significantly correlated with each other, an unplanned analysis was conducted to assess the independent association of each subscale with future behavioral engagement. In unadjusted and adjusted analyses, *Experiential Engagement* was significantly associated with future behavioral engagement (OR_adj_=1.19, 95% CI 1.05-1.34, *P*=.006). In unadjusted and adjusted analyses, *Behavioral Engagement* was not significantly associated with future behavioral engagement (OR_adj_=1.31, 95% CI 0.38-4.59, *P*=.67).

In unadjusted and adjusted analyses, asking users about how engaging they thought the app was did not significantly predict future behavioral engagement (OR_adj_=1.34, 95% CI 0.98-1.84, *P*=.07). In unadjusted and adjusted analyses, asking users about how much they liked the app significantly predicted future behavioral engagement (OR_adj_=1.38, 95% CI 1.03-1.84, *P*=.03).

**Table 5:** Unadjusted and adjusted odds ratios for the associations between the predictor variables and future behavioral engagement.

Predictor variables		Odds ratio (95% CI)	*P* value	Adjusted odds ratio^a^ (95% CI)	*P* value
**Total DBCI^b^ Engagement Scale score**		1.15 (1.05-1.27)	.005	1.14 (1.03-1.27)	.009
	Subscale 1 - Experiential Engagement	1.19 (1.06-1.34)	.004	1.19 (1.05-1.34)	.006
	Subscale 2 - Behavioral Engagement	1.11 (0.90-1.36)	.34	1.08 (0.87-1.35)	.48
“How engaging was the app?”		1.28 (0.96-1.71)	.097	1.34 (0.98-1.84)	.07
“How much did you like the app?”		1.39 (1.05-1.83)	.02	1.38 (1.03-1.84)	.03

^a^Odds ratios adjusted for motivation to reduce alcohol consumption.

^b^DBCI: digital behavior change intervention.

#### Incremental Validity

Results from the incremental validity analyses are reported in Table 6. The automatically recorded behavioral indicators of engagement (ie, items 11 and 12; Model 1) accounted for 15.9% of variance in the variable *subsequent login*. The automatically recorded behavioral indicators in combination with the self-reported experiential indicators of engagement (ie, items 1, 2, 3, and 6; Model 2) accounted for 21.1% of variance in the variable *subsequent login*.

**Table 6:** Odds ratios for the associations between the predictor variables and future behavioral engagement.

Models		Odds ratio (95% CI)	*P* value	Variance accounted for (%)
**Model 1**				**15.9**
	Objectively recorded amount of use	3.46 (1.58-7.57)	.002	
	Objectively recorded depth of use	0.91 (0.58-1.42)	.67	
**Model 2**				**21.1**
	Objectively recorded amount of use	2.86 (1.25-6.55)	.013	
	Objectively recorded depth of use	0.95 (0.60-1.50)	.82	
	Interest	1.72 (1.03-2.85)	.04	
	Focus	0.82 (0.50-1.35)	.44	
	Enjoyment	0.93 (0.61-1.40)	.72	
	Intrigue	1.17 (0.78-1.76)	.45	

#### Divergent Validity

Total scale scores were significantly correlated with the first (“When using *Drink Less*, the way time passed seemed different from normal”) but not the second (“When using *Drink Less*, I was not worried about what others may have been thinking about me”) indicator of flow (*r*_145_=0.25, *P*<.01 and *r*_145_=–0.01, *P*=.95, respectively). The two items tapping flow were not significantly correlated with one another in this sample (*r*_145_=–0.06, *P*=.47).

## Discussion

### Principal Findings

The DBCI Engagement Scale was found to be underpinned by two, largely independent factors, which were labelled *Experiential Engagement* and *Behavioral Engagement.* The scale showed moderate internal reliability, but low divergent and criterion validity. Importantly, the behavioral subscale may not be a valid indicator of behavioral engagement. Total scale scores were weakly associated with future behavioral engagement (ie, the variable *subsequent login*), as were the experiential subscale and one of the *best bets* for a short measure of engagement (ie, asking participants about how much they liked the app). The behavioral subscale was not independently associated with future behavioral engagement. In addition, a model including the self-reported experiential and objectively recorded behavioral indicators of engagement (as compared with a model including only the objectively recorded behavioral indicators) accounted for a larger proportion of variance in future behavioral engagement. These findings are at odds with those from the first evaluation of the DBCI Engagement Scale, in which the scale was found to be underpinned by a single factor [14]. However, these differences may at least partly be accounted for by the small sample size in this study.

### Construct Validity

The finding that the *Experiential Engagement* and *Behavioral Engagement* subscales were not significantly correlated with each other in this study lends support to the argument that users can spend time on a DBCI without necessarily being interested in or paying attention to its content, and vice versa [14]. However, this finding also gives rise to the question of whether experiential and behavioral engagement are part of the same higher-order construct.

The finding that participants’ total scale scores were weakly associated with future behavioral engagement even when adjusting for motivation to reduce alcohol consumption serves as initial evidence that the state of engagement with a DBCI is conceptually distinct from motivation to change the target behavior.

### Incremental and Predictive Validity

The results from the incremental validity analyses suggest that behavioral and experiential indicators in tandem have superior predictive power compared with the behavioral indicators alone. However, the finding that the experiential, but not the behavioral, subscale was independently associated with future behavioral engagement can be interpreted to suggest that the experiential indicators (particularly users’ interest) were driving the association between initial and future engagement. A potential explanation for these findings is that more intensive engagement during the first login session might have made users’ memory of the app more salient, which might have made them more likely to remember to return to the app. As one of the short measures of engagement (ie, the item asking about how much users liked the app) was also found to predict future engagement, it is possible that not only salience of the app, but a salient memory of liking the app, is important for future engagement. It is unclear why the first, but not the second, short measure of engagement had significant predictive power; the word *liking* might be easier to interpret than the word *engaging*. The potential mechanisms underlying the relationship between initial experiential and behavioral engagement, and future behavioral engagement (ie, the variable *subsequent login*) should be explored further using experience sampling techniques in the first few hours following initial app engagement; this involves repeated measurements of psychological processes in real time, in users’ natural environments [36].

These results also beg the question as to whether future behavioral engagement is the most appropriate criterion variable to test an engagement scale against. For example, knowledge retention or skill acquisition may be more theoretically sound, as suggested by the Elaboration Likelihood Model of Persuasion (ELMP) [37]. The ELMP argues that deep information processing occurs when an individual pays attention to (or engages with) a health message, which leads to increased knowledge retention. It is plausible that initial behavioral and experiential engagement have superior predictive power compared with behavioral engagement when used to predict knowledge retention. In addition, it would be useful to assess whether the new measure of moment-to-moment (or state-like) engagement is able to predict intervention effectiveness at a later time point.

### Criterion Validity

The finding that the self-reported and objectively recorded indicators of *amount of use* were not significantly correlated in this sample suggests that the DBCI Engagement Scale may not be a valid indicator of behavioral engagement. However, although the *amount of use* (ie, time spent in minutes or seconds) is typically used as a gold standard or ground truth of behavioral engagement, our results showed that objectively recorded *amount of use* was significantly correlated with many of the experiential indicators (eg, interest, intrigue). Although the exploratory factor analyses did not indicate that *amount of use* loads onto the same factor as the experiential indicators of engagement, the observed pattern of correlations leads us to question whether time spent on a DBCI is deserving of its ground truth status. There is, hence, a need for future research to investigate the source of the discrepancy between self-reported and objectively recorded indicators of *amount of use*.

### Divergent Validity

In line with the first study evaluating the scale, this study did not provide evidence that the DBCI Engagement Scale diverges from the Flow State Scale. There is conceptual overlap between engagement with DBCIs and the dimension of flow that is labelled *losing track of time*. It should be noted that the proposed definition of engagement was, in part, developed based on the concept of flow [14]. It may hence be more fruitful to assess the scale’s divergent validity using a more conceptually distinct measure in the future. The lack of evidence that the DBCI Engagement Scale diverges from the Flow State Scale may also serve as a plausible explanation for why participants’ self-reported *amount of use* was not significantly correlated with their objectively recorded *amount of use*; they may have lost track of time when engaging with the *Drink Less* app. This finding suggests that self-reported and objectively recorded indicators of time spent on a DBCI may tap different constructs; future research is required to examine which of these is more strongly related to key outcomes of interest.

### Limitations

This study was limited because it did not achieve the desired sample size of 250 participants. As Prolific is a novel platform with a small proportion of individuals meeting the study eligibility criteria (ie, drinking alcohol excessively, willing to download an alcohol reduction app, owning an iPhone), the extant participant pool was exhausted after screening just over 400 participants. Although the participant-to-item ratio is considered key in determining the minimum necessary sample size for conducting factor analyses, findings from simulation studies indicate that other factors, including the number of items per factor and the level of communality between items, also influence sample size requirements [38]. Given the limited participant-to-item ratio and the small number of items per factor in this study, the two-factor solution should be interpreted with caution and merits replication in a larger sample in future research. A second limitation is that market research indicates that iOS users are, on average, more affluent than Android users [39]. As the *Drink Less* app is currently available for iOS users only, our findings may not be generalizable to Android users.

Studies conducted via Prolific that involve an initial screening study followed by inviting eligible participants to complete the actual study tend to have attrition rates of approximately 20%-25%, and not 45% [40]. It is therefore likely that there were systematic differences between eligible participants who completed the task and those who did not. For example, the small financial reward may not have been perceived as worth the effort of downloading an app. Indeed, a study assessing the demographic and psychological characteristics of participants who regularly complete research tasks via Amazon’s Mechanical Turk online platform (which is similar to Prolific) found that the majority of surveyed participants reported that earning money was a key motivator for taking part [24]. It should also be noted that the financial incentive may have interfered with participants’ naturalistic engagement, thus limiting the generalizability of the findings. Previous research has found that money can be an important motivator in DBCI research and increase response rates in longitudinal studies [41].

We did not want to overburden users; hence, we did not assess key trait-like variables that may have influenced users’ scale scores. For example, it would have been useful to attempt to partial out the variance accounted for by users’ personality traits, such as those specified in the Big Five model of personality [42], to ensure that the DBCI Engagement Scale is detecting something beyond high conscientiousness or low neuroticism.

The adjustment for participants’ motivation to reduce their alcohol consumption should have increased the item covariance on the DBCI Engagement Scale and is hence considered a study strength. It should, however, be noted that participants’ motivation may have interacted with their engagement levels. Hence, despite the adjustment for participants’ motivation to change, the scale scores may not fully represent participants’ “true” engagement scores.

Finally, the decision to use Google’s cutoff (ie, 30 minutes of inactivity) to identify whether users had made a subsequent login is, to our best knowledge, not grounded in evidence about session length. Future research should explore whether this constitutes a useful heuristic for identifying new DBCI sessions using both quantitative and qualitative methods.

### Avenues for Future Research

Due to the observed nonnormal distributions of the scale items that jointly form the DBCI Engagement Scale, a decision was made to use z-score normalization. Consequently, total scores on the DBCI Engagement Scale are only meaningful in relation to the average intensity of experiential and behavioral engagement that a particular DBCI generates. This may facilitate attempts to develop cutoffs for “high” and “low” engagers across DBCIs, irrespective of their specific parameters (eg, the number and length of intervention components). For example, users with scores that fall within a particular range of SDs above or below the mean might usefully be classified as “high” or “low” engagers, and these patterns may replicate across DBCIs. The question of whether the mean and spread of engagement scores replicate across DBCIs merits exploration by evaluating the DBCI Engagement Scale across different kinds of DBCIs (eg, websites or apps for smoking cessation or physical activity).

The finding that initial experiential engagement (or liking of the app) was independently associated with future behavioral engagement suggests that intervention developers should think carefully about how to make their DBCIs appealing on first use. The DBCI Engagement Scale may be useful during the iterative design process, comparing users’ experiences of differently designed graphical user interfaces.

### Conclusions

The DBCI Engagement Scale assesses behavioral and experiential aspects of engagement. The behavioral subscale may not be a valid indicator of behavioral engagement. The experiential subscale can predict subsequent behavioral engagement with an app for reducing alcohol consumption. Further refinements and validation of the scale in larger samples and across different DBCIs are needed.

## Abbreviations

AUDIT: Alcohol Use Disorders Identification Test
DBCI: digital behavior change intervention
